# The Effect of Social Media Scrolling on Working Memory Among Indian Medical Students: A Prospective Comparative Observational Study

**DOI:** 10.7759/cureus.105147

**Published:** 2026-03-13

**Authors:** Ayush Mittal, Anushka Sohu, Vibhuti Agarwal, Souvik Manna, Sumit Grover

**Affiliations:** 1 Medicine, ESIC Medical College and Hospital, Alwar, IND; 2 Biochemistry, ESIC Medical College and Hospital, Alwar, IND; 3 Community Medicine, ESIC Medical College and Hospital, Alwar, IND; 4 Ophthalmology, National Cancer Institute, All India Institute of Medical Sciences (AIIMS) New Delhi Jhajjar Campus, New Delhi, IND; 5 Volunteer, Manthan Eye Healthcare Foundation, Gurgaon, IND

**Keywords:** attention deficit disorder, digit span test, effects of social media, internet addiction disorder, working memory deficits

## Abstract

Background and objective

Social media use among university students, especially medical students, has become widespread, and frequent use has been linked to cognitive distractions and potential deficits in attention and memory. Working memory is a type of short-term memory and a core executive function that temporarily stores and manipulates information, and is particularly vulnerable to disruption from the frequent use of highly engaging social media content. Most existing literature focuses on the association between prolonged social media usage and cognitive decline. However, very few experimental studies have evaluated the immediate effects of social media scrolling on working memory. This comparative observational study examined whether recent social media scrolling (within 30 minutes) impairs working memory among Indian medical students.

Methods

It was hypothesized that students who scrolled through social media for more than 30 minutes would perform worse on the digit span working memory test than their peers engaged in non-digital tasks. A total of 140 participants (mean age: 20.5 years) were recruited, of which 58 (41.4%) were males, 124 (88.6%) were residents of Rajasthan, 136 (97.1%) identified as Hindu, 139 (99.2%) lived in hostels, 67 (47.8%) reported an annual family income of less than five lakhs, and around half of the participants (71, 50.7%) belonged to nuclear families. Participants from both genders were equally represented across each year of study. All the participants were divided into a social media group (n = 70) and a control group (n = 70). The social media group completed the digit span forward and backward tests immediately after 30 minutes of social media scrolling, whereas the control group completed the same tests after engaging in a non-digital activity. The mean forward and backward digit span scores were calculated across different sociodemographic variables.

Results

The social media group scored lower than the control group on both forward (6.63 ± 1.30 vs. 7.61 ± 1.77) and backward (5.46 ± 1.51 vs. 5.90 ± 1.79) digit span tests (p < 0.001 and p = 0.118, respectively). Forward digit span scores differed significantly between age groups, with younger participants demonstrating higher scores (F = 3.80, p = 0.02). Backward digit span scores varied significantly according to year of study (F = 3.76, p = 0.02), residence (t = −2.30, p = 0.02), and religion (F = 2.76, p = 0.04).

Conclusions

Students who engage in social media usage, especially with short-form video content, demonstrate poorer working memory performance. These findings also suggest that even short-term social media usage may acutely impair working memory capacity. This indicates potential negative cognitive effects of social media usage, including reduced focus and attention among students.

## Introduction

Social media use among university students, especially medical students, has become ubiquitous. While these platforms offer avenues for connection and information, their frequent use has been associated with cognitive distractions and potential deficits in attention and memory. The era of digital communication has led to decreased face-to-face interactions, which may affect attention span and focus [1]. A study found that 63.76% of medical students exhibited mild social media addiction, with 36.23% classified as having moderate addiction [2]. It has also been reported that excessive social media use correlates with increased forgetfulness and distractibility, thereby negatively affecting cognitive functions [3]. In individuals with attention deficit hyperactivity disorder, exposure to social media was associated with a notable decline in attention span, with 60% of participants reporting attention spans of less than 60 seconds after 30 days of use [4].

Working memory, a type of short-term memory, is a core executive function that temporarily stores and manipulates information. It is crucial for academic performance and clinical decision-making in medicine. Recent studies have investigated the impact of social media usage on academic performance and working memory among university students, particularly medical students. Some of these studies found a negative correlation between social media use and academic performance [5]. Research on Chinese high school students showed that frequent social media use decreased their ability to concentrate on academic tasks [6], although the effects differ among individuals. In another study, students reported that social media scrolling disrupted their study hours, leading to poor academic performance and frequent class absenteeism [7]. The rise of platforms like YouTube Shorts and Instagram Reels has been linked to shorter attention spans, with studies showing a significant negative correlation between time spent on short-video apps and attention span in young adults (r = -0.404) [8].

Most existing literature focuses on the association between prolonged social media usage and cognitive decline. However, only a few experimental studies have assessed the immediate effects of social media scrolling on working memory, particularly among Indian medical students, who experience considerable cognitive load due to a demanding academic curriculum. The current study aimed to determine the short-term impact of social media usage on the cognitive functioning of medical students by assessing its effect on working memory. The study also sought to compare working memory performance between students engaged in social media and those performing non-digital tasks.

## Materials and methods

The study employed a prospective comparative observational design, and the study setting was ESIC Medical College and Hospital, Alwar, Rajasthan, Western India. It was conducted over one month in November and December 2025. The study population consisted of 140 MBBS students from the first three phases, aged 17 to 27 years.

A sample size of 140 participants (70 in each group) was calculated using OpenEpi software, assuming a 95 confidence interval (CI), an equal ratio of exposed to unexposed, 50% outcome among unexposed, 75% outcome among exposed, and a 5% non-response rate. The sampling technique was stratified random sampling (stratified by year of study and gender). The sampling and stratification were conducted to ensure an equal number of participants of each gender in both groups while also maintaining proportional representation from each year of study in both groups.

The inclusion criteria required MBBS students to be aged 17 to 27 years and to be either daily social media users (regular use) or occasional users (less than one hour per week). Only students who provided informed consent were recruited. Daily users were assigned to the social media group, while occasional users were assigned to the non-social media group. Students diagnosed with attention deficit or other psychiatric disorders, currently taking medications affecting cognition, or with a history of substance abuse were excluded from the study. The null hypothesis (H₀) stated that there was no significant difference in working memory performance between students who scroll social media and those who do not.

To assess working memory, the digit span test was used, which is a widely accepted neuropsychological test that measures the ability to store and manipulate numerical information over short periods and is commonly included in intelligence and cognitive assessments. A computerized digit span forward and backward task, adapted from the Wechsler Adult Intelligence Scale (WAIS) paradigm [9], was administered using the Psychology Experiment Building Language (PEBL) test battery [10]. The task involved auditory presentation of digit sequences with increasing length, requiring immediate serial recall. It was performed in two forms: the digit span forward test, in which participants were asked to repeat sequences of numbers in the same order as presented to assess short-term memory and attention, and the digit span backward test, in which participants were asked to repeat the digits in reverse order. The backward test specifically required working memory, as it involved both storage and manipulation of information.

Students from the first three years of the medical college were recruited for the study, and both digit span tests (forward and backward) were administered. Participants were stratified according to their daily average screen time into two groups: the social media group, consisting of students under objective monitoring who had scrolled for more than 30 minutes in the preceding hour on platforms such as Instagram™, Facebook™, and YouTube™, and the non-social media group, consisting of students who had not scrolled or had scrolled for less than 30 minutes in the preceding hour, preferably engaged in non-digital activities such as reading magazines or neutral articles.

The data were kept anonymous, and confidentiality was maintained in accordance with GCP guidelines. Informed consent was obtained digitally through a Google Forms-based consent sheet, which provided a clear explanation of the study’s purpose, procedures, minimal risks, and voluntary nature. Participants were required to actively agree by selecting a mandatory consent checkbox before proceeding to the questionnaire. No personal identifiers were collected, ensuring complete anonymity. Only participants who provided consent were able to access and complete the study form. All responses were securely stored in Google Forms with access restricted to the research team. The right to withdraw from the study at any time was fully respected.

Statistical data analysis was done using Jamovi software (version 2.7.13.0). Sociodemographic variables like age, gender, religion, place of residence, accommodation type, family type, and annual family income were used for comparing means between forward and backward tests. Categorical variables were reported as percentages and continuous variables as mean (and standard deviations (SD). An independent sample t-test was done to compare two means, and a chi-square test was done to compare two proportions. All reported p-values were two-tailed; statistical significance was set at 0.05 probability. Additional statistical tests, such as Pearson correlation, simple linear regression, coefficient of determination (r^2^), stratified analyses, and ANOVA F-test were also performed.

## Results

The current study included a total of 140 participants, of whom 58 (41.4%) were males, 124 (88.6%) were locals (from Rajasthan state), 136 (97.1%) were Hindu, 139 (99.2%) were hostellers, and 67 (47.8%) had an annual family income of less than five lakh. Around half of the participants (71, 50.7%) belonged to nuclear families, and equal numbers of participants of each gender were selected from each year of study. The social media group (n = 70) and the control group (n = 70) were similar with respect to age (t-statistic = -0.82, p = 0.416) and had a comparable gender distribution (χ² = 0.11, p = 0.732) (Table 1).

**Table 1:** Demographic variables of study participants by comparison group (N = 140) SD: standard deviation

Characteristic	Social media group (n = 70)	Control group (n = 70)
Age, years, mean (SD)	20.4 (1.5)	20.6 (1.4)
Gender, n (%)		
Male	30 (42.9%)	28 (40.0%)
Female	40 (57.1%)	42 (60.0%)
Year of study, n (%)		
First year	32 (45.7%)	32 (45.7%)
Second year	19 (27.1%)	19 (27.1%)
Third year	19 (27.1%)	19 (27.1%)

Mean forward and backward digit span scores across different sociodemographic variables were calculated. Forward digit span scores differed significantly across age groups, with younger participants demonstrating higher scores (F = 3.80, p = 0.02). Backward digit span scores showed significant variation by year of study (F = 3.76, p = 0.02), residence (t = −2.30, p = 0.02), and religion (F = 2.76, p = 0.04). No statistically significant differences were observed in digit span scores across gender, accommodation type, family income, or family structure (p > 0.05). Overall, backward digit span demonstrated greater sensitivity to sociodemographic factors than forward digit span (Table 2).

**Table 2:** Mean scores based on demographic details of the study participants (N = 140) ^*^Statistically significant SD: standard deviation; F-stat: ANOVA statistics; p-value: probability value; t-stat: independent sample t-statistics

Mean scores based on demographic details			
Age group, years	Forward mean (±SD)	Backward mean (±SD)	N (%)
17-19	7.57 (±1.39)	5.78 (±1.86)	40 (28.57)
20-22	7.03 (±1.69)	5.67 (±1.60)	90 (64.28)
Above 23	6.10 (±1.37)	5.40 (±1.50)	10 (7.14)
F-stat, p-value	3.80, 0.02^*^	0.21, 0.80	
Gender			
Male	7.19 (±1.69)	5.71 (±1.65)	58 (41.42)
Female	7.07 (±1.58)	5.66 (±1.67)	82 (58.57)
t-stat, p-value	0.43, 0.66	0.17, 0.86	
Year of study			
First-year student	7.34 (±1.63)	5.50 (±1.70)	64 (45.71)
Second-year student	7.16 (±1.42)	6.29 (±1.76)	38 (27.14)
Third-year student	6.71 (±1.75)	5.37 (±1.32)	38 (27.14)
F-stat, p-value	1.84, 0.16	3.76, 0.02^*^	
Residence			
Inside Rajasthan	7.10 (±1.64)	5.56 (±1.59)	124 (88.57)
Outside Rajasthan	7.25 (±1.52)	6.56 (±1.93)	16 (11.42)
t-stat, p-value	-0.34, 0.72	-2.30, 0.02^*^	
Religion			
Hindu	7.10 (±1.64)	5.62 (±1.62)	136 (97.14)
Muslim	8.00 (±0.0)	6.00 (±0.0)	1 (0.71)
Sikh	8.00 (±0.0)	8.50 (±2.12)	2 (1.42)
Christian	8.00 (±0.0)	8.00 (±0.0)	1 (0.71)
F-stat, p-value	0.39, 0.75	2.76, 0.04^*^	
Accommodation			
Hostel single room	6.82 (±1.47)	5.91 (±1.57)	9 (6.42)
Hostel shared room	7.15 (±1.64)	5.64 (±1.66)	130 (92.85)
Home	7.00 (±0.0)	8.00 (±0.0)	1 (0.71)
F-stat, p-value	0.17, 0.83	1.10, 0.33	
Annual family income			
< 5 Lakhs	7.06 (±1.76)	5.42 (±1.46)	67 (47.85)
5-8 Lakhs	7.20 (±1.37)	5.66 (±1.82)	44 (31.42)
8-12 Lakhs	7.13 (±1.62)	6.06 (±1.52)	16 (11.42)
> 12 Lakhs	7.15 (±1.81)	6.62 (±1.93)	13 (9.28)
F-stat, p-value	0.07, 0.97	2.29, 0.08	
Family type			
Joint	7.33 (±1.67)	5.47 (±1.68)	64 (45.71)
Nuclear	6.97 (±1.60)	5.89 (±1.63)	71 (50.71)
Extended	6.60 (±1.14)	5.40 (±1.81)	5 (3.57)
F-stat, p-value	1.09, 0.33	1.15, 0.31	

Participants in the social media group had spent more than 30 minutes on social media just before testing, whereas the control group had engaged in the non-digital task of reading a literary article. Both groups then completed the forward and backward digit span tests. It was found that the forward memory scores of the social media group were significantly lower compared to the controls on the forward digit span test, but not on the backward test (Table 2). Specifically, the social media group’s forward digit span was 6.63 ± 1.30 digits, compared with 7.61 ± 1.77 digits in controls (t = -3.73, p = 0.000); the backward span was 5.46 ± 1.51 digits compared with 5.90 ± 1.79 digits, respectively (t = -1.57, p = 0.118) (Table 3).

**Table 3:** Comparison of mean digit span test scores among the two group (N = 140) SD: standard deviation

	Group	N	Missing	Mean	Median	Mode	SD	Range	Minimum	Maximum
Backward digit span	Social media	70	0	5.46	5.00	5.00	1.51	7	2	9
	Non-social-media	70	0	5.90	6.00	7.00	1.79	8	2	10
Forward digit span	Social media	70	0	6.63	7.00	7.00	1.30	6	4	10
	Non-social-media	70	0	7.61	8.00	8.00	1.77	9	2	11

These results indicate that acute social media scrolling was associated with a significant decrease in short-term working memory performance. No floor or ceiling effects were noted, and scores varied widely (e.g., forward span ranged from 3 to 12 digits). No adverse events occurred, and test administration was completed successfully for all participants. The homogeneity of variance was tested using Levene’s test, which indicated equal variance in both comparison groups, indicating the parametric nature of the data. Given the similar demographics and the standardized testing procedure, an association can be inferred between recent social media exposure and lower memory scores in the social media group compared to the control group.

**Figure 1:**
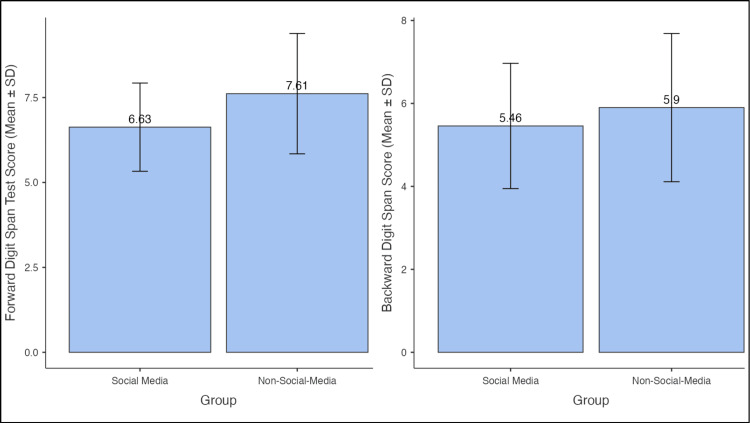
Forward and backward digit span test scores comparison between social media and non-social-media groups (N = 140) 1. The Jamovi project (2025). Jamovi (Version 2.7). Retrieved from https://www.jamovi.org. 2. R Core Team (2025). R: A Language and environment for statistical computing. (Version 4.5). Retrieved from https://cran.r-project.org. (R packages retrieved from CRAN snapshot 2025-05-25) SD: standard deviation

Students who had recently engaged in social media scrolling scored lower on both forward and backward digit recalls compared to those who engaged in non-digital reading tasks. The deviation bars (standard deviations) indicate that the dispersion of the groups is consistent and statistically meaningful, with the non-social-media group demonstrating a statistically higher working memory score on these measures. Since the backward digit span relies more heavily on active working memory manipulation, the decline further illustrates the acute negative effect of social media scrolling on cognitive functioning. The bar diagram below shows a clear separation between the two groups (Figure 1).

**Figure 2:**
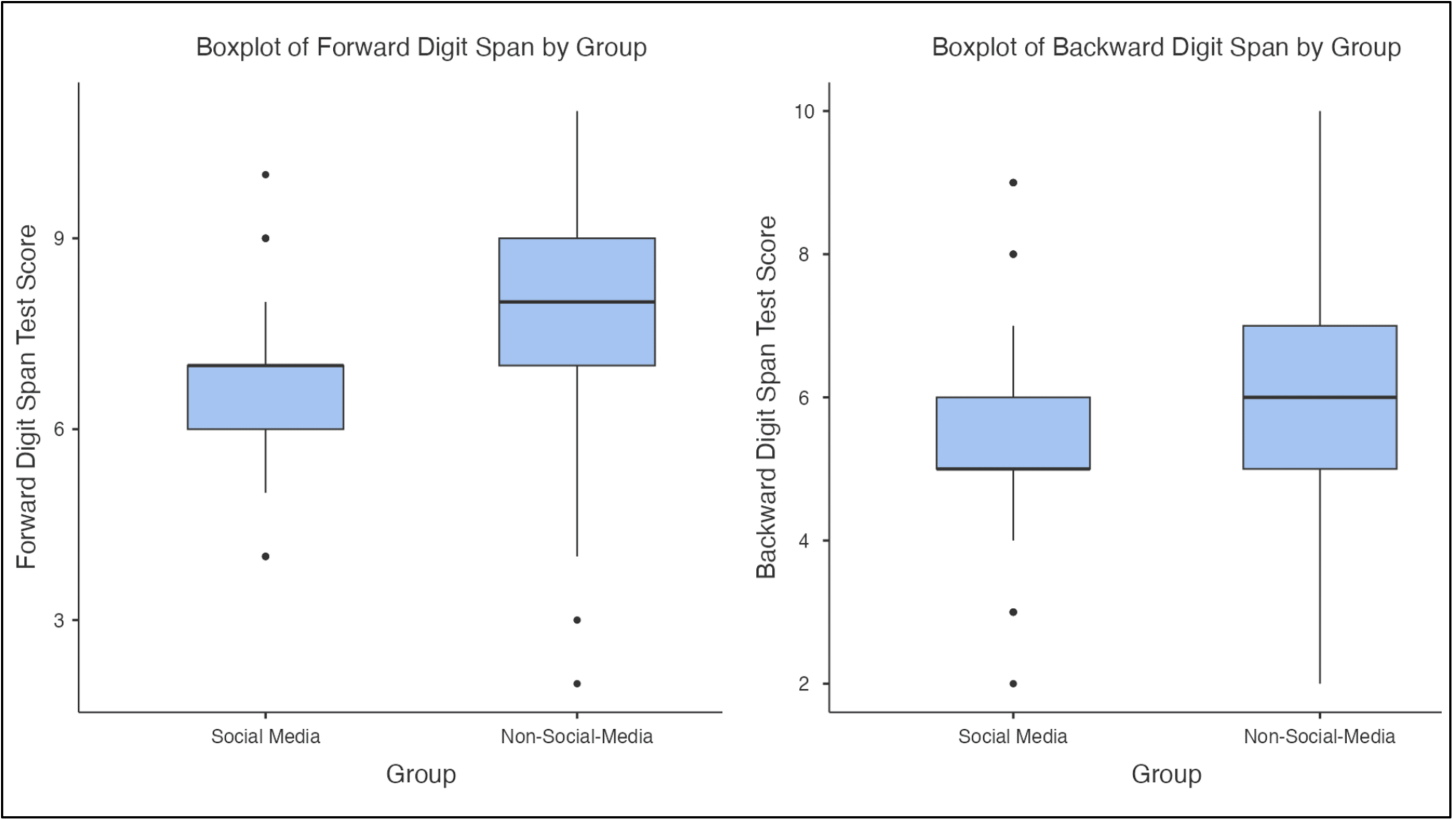
Box and whisker plots of forward and backward digit span test scores segregated by social media groups (N = 140) 1. The Jamovi project (2025). Jamovi (Version 2.7). Retrieved from https://www.jamovi.org. 2. R Core Team (2025). R: A Language and environment for statistical computing. (Version 4.5). Retrieved from https://cran.r-project.org. (R packages retrieved from CRAN snapshot 2025-05-25)

The non-social media group showed a higher forward test median score and a generally higher interquartile range, indicating better performance overall. The social media group, on the other hand, demonstrated a lower median and reduced spread, with several low-end scores appearing as outliers. This visualization emphasizes that the decline in memory performance after social media use is not limited to differences in the mean but is consistent across the distribution. The non-social media group also demonstrated a higher median and broader distribution on the backward test. In contrast, the social media group was clustered around lower values, with multiple low-range outliers. Since backward digit span is a more demanding assessment of working memory, this graph illustrates that the negative cognitive impact of brief social media scrolling is particularly evident in tasks requiring higher mental effort (Figure 2).

**Figure 3:**
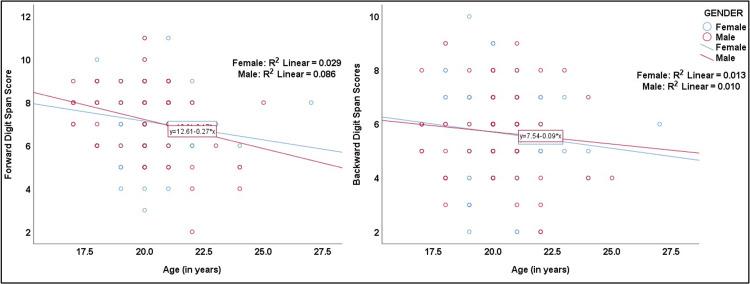
Scatter plots showing the association of age with forward and backward digit span scores stratified by gender (N = 140) R^2^: coefficient of determination

Scatter plots illustrated a negative correlation between age and forward and backward digit span scores when stratified by gender, with fitted interpolation lines. The coefficients of determination (R²) showed that age explained only a small proportion of the variance in digit span scores, with a slightly stronger decline observed for forward digit span compared to backward digit span. Among males, 8.6% and 1% of the variance in forward and backward digit span scores, respectively, were explained by age, whereas among females, 2.9% and 1.3% of the variance in forward and backward scores were explained by age, respectively. Overall, the age-related trends were minimal and comparable across genders (Figure 3).

## Discussion

The findings of the study demonstrate that brief social media scrolling is associated with impaired working memory among medical students. The significantly lower digit span scores on forward testing among participants exposed to social media suggest an acute cognitive cost of digital distraction. These results support the alternative hypothesis of the study and align with existing literature on attention deficits associated with social media use. Ahmed et al. reported that higher social media engagement corresponded to greater forgetfulness and distractibility among students [3]. Roshan et al. similarly found that daily social media exposure led to 60% of subjects having attention spans of less than one minute, underscoring reduced concentration and ADHD [4]. The observed effect in the current study likely reflects a combination of diverted attention and reduced capacity for rehearsal after scrolling, consistent with the idea that shifting focus to rapid online content disrupts working memory [8,11].

Broadly, the impact of social media on students' cognitive function remains complex and inconclusive. Some studies report no significant association between social media use and cognitive functioning [12], or that when an association exists, the effect is predominantly minor and does not outweigh age and demographic differences [13]. Some studies have even reported possible minor positive effects, such as slightly higher verbal ability among long-term social media users [14]. However, the actual difference may lie in patterns of use, which vary from person to person and may also depend on exposure variables such as age, intelligence, rest, and cognitive ability. The current study also did not demonstrate a significant reduction in the backward test scores compared with the forward test scores.

The results of studies targeting older adults and senior citizens have been more inconsistent, with multiple studies stating that social media usage can potentially improve cognitive domains such as executive function, processing speed, and inhibitory control [15-20]. However, other studies have reported that such usage is associated with cognitive failures [21-24]. Therefore, current research suggests more nuanced cognitive impacts than simple positive or negative outcomes, indicating the need for more sophisticated, longitudinal studies to definitively understand the cognitive effects of social media. In addition, familiarity with social media and the level of technological dexterity might limit social media usage among the elderly population.

The majority of researchers have concluded that long-term or prolonged social media usage negatively affects the cognitive functioning of students [25-28], especially when combined with precipitating factors such as social anxiety and depression [29,30]. In contrast to long-term studies of academic performance [5,7], the current study specifically measured immediate memory performance following scrolling. The strong group differences (p < 0.001) reported suggest a robust effect size on forward testing. This indicates that even short episodes of social media use can impair memory performance, which may accumulate to impact learning if repeated. The results are consistent with findings of shorter attention spans in heavy TikTok™ users [8] and with classroom studies showing that multitasking with social media reduces information recall [11].

No previous studies have directly compared short-term digit span after social media versus non-digital tasks in this population, so the current study is an important addition to the literature. It suggests that the active mental engagement of scrolling, likely characterized by frequent switching and stimulus novelty, may momentarily occupy working memory resources. This is in line with theories of cognitive overload, where rapid media inputs can saturate short-term memory capacity and leave fewer resources for new information. The study design (immediate testing after one hour) reflects a worst-case scenario of cognitive loading, which is realistic for students who take study breaks to browse social feeds.

This study has a few limitations. Firstly, the quasi-experimental design meant that participants were not randomly assigned; students were grouped based on their recent behavior. It is possible that inherently different students chose to scroll or not, although stratification by year of study and gender may have reduced this bias. Baseline cognitive ability or sleep, which were not measured, could have confounded the results. Second, digit span tests assess only one aspect of working memory (verbal span); other functions, such as visual memory or complex span tasks, may show different patterns. Third, the study reflects an acute effect, and it is unclear whether frequent short-term deficits translate into long-term cognitive decline or adaptive changes. A longer study with repeated follow-ups would be better suited to evaluate long-term deficits or learning impairment resulting from social media usage.

Additionally, the small sample, limited to one medical college in North India and comprising young participants aged 17-27 years, restricts the external validity of the study. Cultural and curricular differences may influence the generalizability of the findings to other academic streams or regions. Future research should include randomized crossover designs, objective monitoring of social media use, and potentially neurophysiological measures (e.g., EEG of attention) to clarify underlying mechanisms. Longitudinal studies could investigate whether repeated short-term impairments accumulate or whether students develop compensatory strategies over time. Intervention studies, such as mindfulness training to reduce distraction, could explore protective approaches.

## Conclusions

This study concluded that the social media group had lower working memory scores compared to the non-social media group. Acute social media scrolling, therefore, caused immediate attention disruption. However, backward digit span showed a non-significant decline, likely because it requires a higher cognitive load. This study provides evidence that acute social media scrolling can immediately impair working memory performance in medical students. Specifically, after more than 30 minutes of social media use, participants recalled significantly fewer digits than peers engaged in non-digital tasks. These results align with existing literature linking digital media to attention and memory problems and suggest practical implications for study habits. Given that working memory underpins learning and clinical decision-making, students and educators should recognize that even brief media distractions may reduce cognitive performance. In summary, acute social media use was associated with a short-term decline in working memory, highlighting the need for moderation in media scrolling, particularly during study or clinical preparation.
